# Duty of care in companion dog owners: Preliminary scale development and empirical exploration

**DOI:** 10.1371/journal.pone.0285278

**Published:** 2023-05-17

**Authors:** Carmen R. Glanville, Lauren M. Hemsworth, Paul H. Hemsworth, Grahame J. Coleman

**Affiliations:** Animal Welfare Science Centre, Faculty of Veterinary and Agricultural Sciences, University of Melbourne, Melbourne, Victoria, Australia; St John’s University, UNITED STATES

## Abstract

Owner behaviour change may be the most promising avenue to improve dog welfare. As such, understanding potential drivers of owner behaviour is critical to developing effective intervention programs. Here we examine in depth the concept of “duty of care” as a motivator of owner behaviour. Using a mixed methods approach, this study aimed to better understand the potential dimensions of duty of care, their interrelationships, and develop psychometrically valid tools to measure them in companion dog owners. This was achieved through a multi-stage process including a critical literature review, qualitative interviews (*n* = 13), and an online survey (*n* = 538). Using Schwartz’s Norm Activation Model as a framework, we have developed a 30 item scale with 5 subscales; duty beliefs, problem awareness, awareness of impact, efficacy, and ascription of responsibility. These unique subscales demonstrate good internal consistency and construct validity. In addition to developing a measurement tool, this process has provided important insights into the nature of duty of care in companion dog owners, creating several avenues for future inquiry. One such finding was that many dog welfare problems may not be the result of lacking duty beliefs, but rather weaknesses in other “activation” factors such as problem awareness or ascription of responsibility. Further work is now required to understand the predictive validity of the scale and the relative influence of its different dimensions on owner behaviour and dog welfare outcomes. This will facilitate the identification of appropriate targets for intervention programs aimed at improving owner behaviour and consequently, dog welfare.

## Introduction

**Fig 1:**
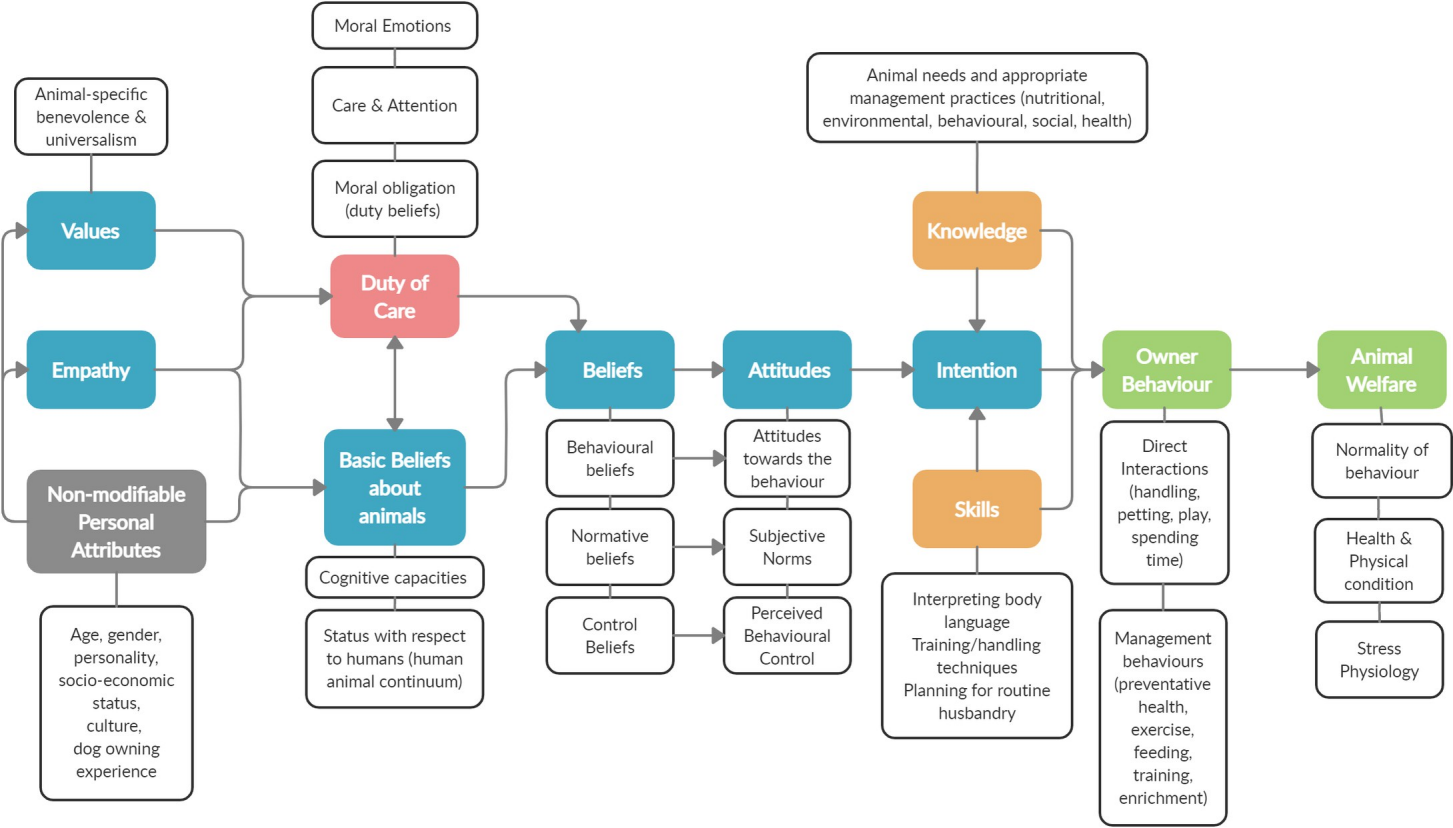
The Pet Care Competency model. Adapted from Glanville et al. [4].

The welfare of companion dogs is most heavily influenced by owner behaviour [1, 2]. As such, human behaviour change is becoming recognised as one of the most promising and important avenues for improving companion dog welfare [3]. To change owner behaviour, it is important to understand and evaluate its various drivers and their relative influence on behaviour. In a previous paper, we presented a new theoretical model of owner behaviour which we called the Pet Care Competency model (PCC, Fig 1) [4]. This new model draws on several well-established, empirically validated psychological theories and builds on the substantial body of human-animal relationship work both with companion animals and in other contexts such as livestock industries and zoo settings.

The original Human-Animal Relationship (HAR) model by Hemsworth and Coleman, demonstrates causal relationships between animal-carer beliefs and attitudes, animal behaviour, and animal welfare [5]. The beliefs and attitudes in the HAR model are drawn from the Theory of Planned Behaviour [6] and are behaviour-specific. That is, if we were interested in predicting dog walking behaviour, the salient attitudes and beliefs would be those related to dog walking specifically. Furthermore, the more specific the beliefs and attitudes that are measured, the more predictive they will be of the target behaviour e.g., if the target behaviour was owners walking their dog twice a day for 30 minutes, then agreement/disagreement with the belief statement “It is important to walk my dog twice a day for 30 minutes” will be a better predictor than the more generic “Walking my dog is important”. While these types of attitudes are considered the most proximate to behavioural intentions and behaviour, their specificity may present a challenge for interventions. This is because there is such a wide range of behaviours required to care for and interact with animals appropriately that it may be difficult to target all of the important factors in a single intervention. This is particularly the case with regards to companion animal ownership as management behaviours are more varied than in commercial settings like livestock farming. As such, with the PCC model we aimed to highlight potential antecedents to these behaviour-specific beliefs and attitudes with the goal of identifying more general motivators that may influence a suite of related behaviours.

One key development of the PCC model was the introduction of ‘duty of care’ as one of these more general (yet still specific enough to maintain predictive validity) motivational constructs. We proposed that duty of care is: “a potentially powerful and unique motivator with both cognitive and affective dimensions, representing the marriage of moral obligations and attentive care” [4, pg. 279]. A multitude of research questions arise from this proposition: what type of owner behaviours are motivated by duty of care and to what extent? Can the same behaviour be motivated by duty of care in one person and by other factors in another? Is behaviour that is motivated by duty of care more enduring or powerful than other more pragmatic or material motivators? In order to study any of these types of questions in a robust way, a thorough understanding of the different elements of duty of care and a validated tool to assess them is required. As such, this study aimed to 1) better understand the potential elements of duty of care and their interrelationships, and 2) develop psychometrically valid tools to assess them in companion dog owners.

## Methods

### Ethics approval

This project was conducted in accordance with the National Statement on Ethical Conduct in Human Research (2007) Guidelines and Regulations. Ethics approval for the interviews was granted by the University of Melbourne Psychology, Health, and Applied Sciences Human Ethics Sub-Committee (project ID: 1953580) and approval for the online survey was provided by the University of Melbourne Human Ethics Advisory Group LNR-2D (project ID: 21548). Prior to engaging, all participants were provided with a Plain Language Statement and completed a consent form. They were advised that they could end their participation at any time and were able to withdraw their data upon completion of the task if they chose to do so.

### Scale development process

**Fig 2:**
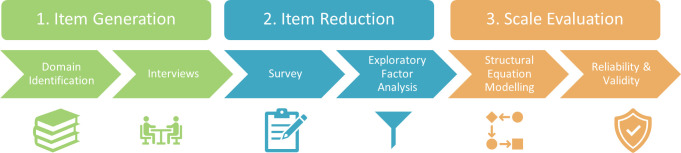
The scale development process. Based on and adapted from [7].

Where practically possible, we followed the “best practice” scale development process outlined in [7]. Fig 2 provides a graphical representation of the process employed in the present study.

### Stage 1: Item generation

The first stage of scale development is to generate a large pool of potential items for inclusion in the scale. This begins with domain identification, followed by more targeted deductive and inductive methods of item generation.

#### Domain identification

Domain identification involves clearly specifying the purpose, boundaries, and *a priori* dimensions of the construct of interest [7]. This is especially important for a novel, primarily theoretical concept like duty of care. As such, we conducted a narrative literature review to build on the preliminary conceptual descriptions of duty of care provided in our previous paper [4].

During this literature review, we found that our original conceptual descriptions of duty of care resonated strongly with Schwartz’s Norm Activation Model (NAM) [8]. The NAM is a psychological model of prosocial behaviour which centres around feelings of moral obligation as the primary motivator. As such, we have adopted this model as a framework for examining the potential dimensions and mechanisms of duty of care and how they may work together to motivate behaviour.

While originally developed for explaining altruistic behaviours, the Norm Activation Model has since been applied to other prosocial behaviours and areas such as pro-environmental behaviours [9], transport choices [10], energy saving [11], recycling [12], and occupational safety [13]. It has often been used in conjunction with the Theory of Planned Behaviour [6], to increase its predictive ability when studying behaviour with a prosocial element e.g., [11, 14–16]. Indeed, Schwartz argued that the model was not exclusive to altruistic behaviour and should apply in any circumstance where behaviour is motivated by moral obligations [17]. In the current context, dog owner care and management behaviour can be considered prosocial in that it is concerned with the welfare of another. However, that is not to say that *all* owner behaviours are motivated by prosocial intentions. Indeed, the same behaviour may arise from different motivations in different people, or even a combination of motivations within the same person. For example, dog walking may be motivated by prosocial intentions of providing for the welfare of the dog, but it may also be motivated by self-serving motivations for exercise, or pragmatic motivations that if the dog isn’t taken for a walk it will become destructive. As such, by examining duty of care in the framework of the NAM, we do not suggest that all dog management behaviour is solely motivated by duty of care. Rather, we aim to understand the role it may have in different behaviours relative to other potential motivators. While obligations or duties towards dog walking have been investigated in two studies [18, 19], to our knowledge the full NAM has yet to be applied to the care and management of companion animals.

Schwartz broadly outlines the NAM in terms of three basic propositions:

1. **Obligations**: prosocial behaviour is influenced by the strength of an individual’s feelings of moral/personal obligation to perform that behaviour
2. **Activation**: situational factors ‘activate’ an individual’s ‘personal norms’ in a specific circumstance, generating these feelings of moral obligation
3. **Defence**: action stemming from feelings of moral obligations may be neutralised by defences regarding the relevance or appropriateness of the obligation.

Beyond these central propositions, the interpretation and practical application of the NAM has been rather inconsistent. There are 5 main inconsistencies we have seen (and which have been identified to differing degrees by others previously [e.g., 20, 21]. In the interest of brevity, we will provide a single example of each:

1. inconsistency in the definitions of key variables (i.e., variables are called the same thing but defined differently), e.g., de Groot and Steg [22] define “awareness of consequences” as “whether someone is aware of the negative consequences for others or for other things one values when not acting prosocially.” (pg 427) while Harland et al. [20] define it as “a person’s receptivity to situational cues of need” (pg 324).
2. inconsistency in the naming of key variables (i.e., variable is defined in the same way but called something different), e.g., what de Groot and Steg [22] defined as “awareness of consequences”, is called “problem awareness” in Steg and de Groot [21].
3. varied use of behaviour-specific versus more general level beliefs, e.g., Onwezen et al. [23] used general beliefs related to “protecting the environment” while Bamberg et al. [10] used more specific beliefs around car use and public transport.
4. different number of variables are used with some key omissions, e.g., Harland et al. [20] use seven of the NAM variables originally outlined by Schwartz, while most studies only use three or four [e.g., 24].
5. the modelled relationship between variables differs, i.e., some use a mediation model [22], some use a moderation model [25], and others use a combination of moderation and mediation [23].

**Fig 3:**
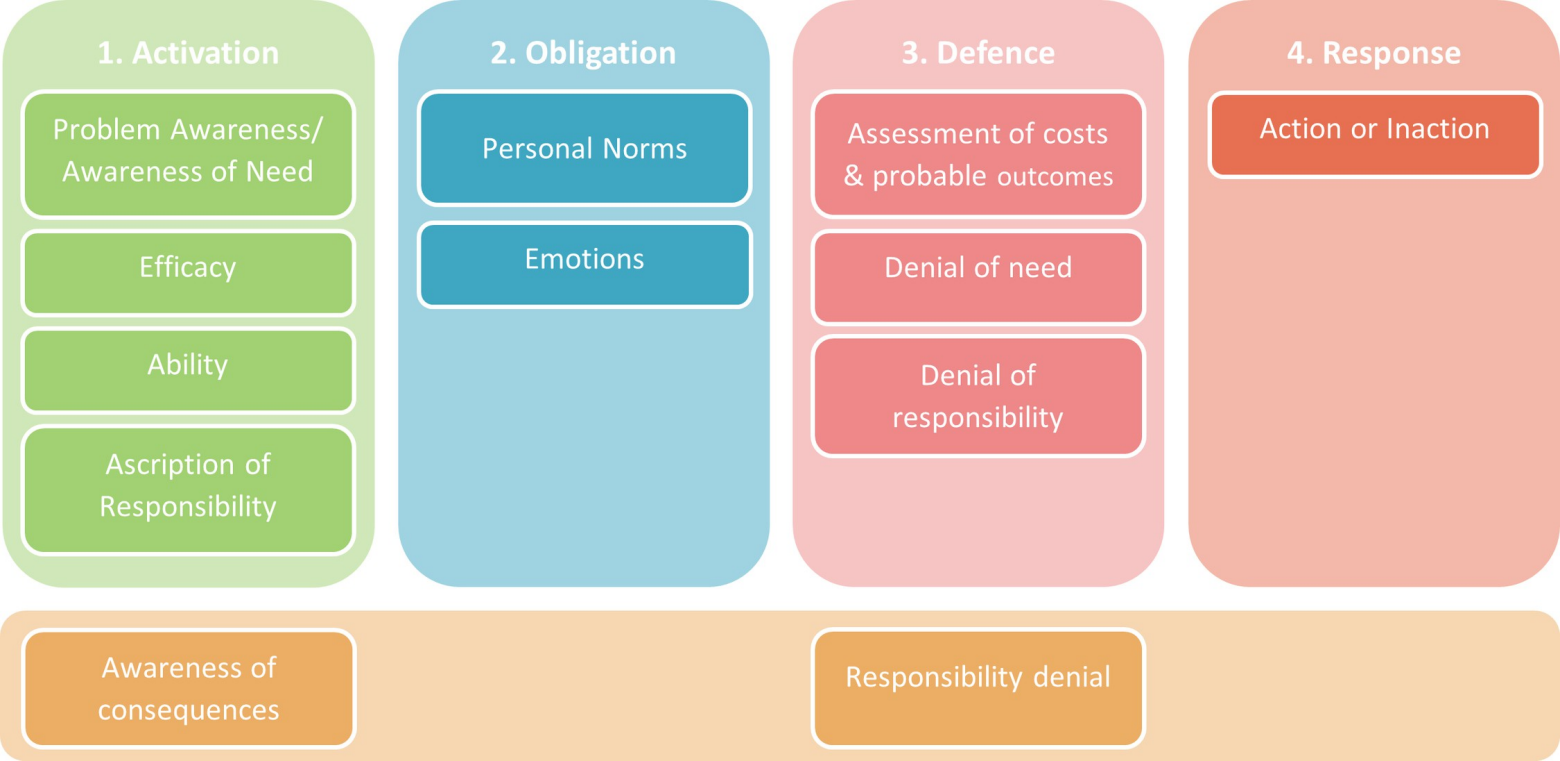
The Norm Activation Model. Key stages and associated situation-specific state variables derived from Schwartz [8]. Trans-situational traits coloured yellow.

It is beyond the scope of this paper to present a detailed analysis of these inconsistencies. As such, in our interpretation of the NAM we have drawn primarily on the original descriptions in Schwartz [8] and Schwartz and Howard [26]. Fig 3 provides a summary of the 4 key stages in the NAM [21] and the associated variables within these steps. Here we have purposely not included arrows to denote the structure of the model as this is an ongoing debate and an empirical question to be addressed later.

In short, the process begins with the activation stage which moves through several steps including the initial *awareness* or recognition of a problem or party in need, followed by the identification of actions that could relieve that need (*efficacy*), belief in one’s *ability* to perform those actions, and finally the sense of *responsibility* to be involved with the needy party. These steps lead to the activation of *personal norms* (defined later) and anticipated *emotions* which generate feelings of *moral obligation* to perform the behaviour. Once the feelings of moral obligation have been generated, the associated action and its outcomes are *assessed* in terms of costs and benefits to the actor. Where costs outweigh the benefits, a range of *defence* mechanisms can be employed to neutralise the feelings of moral obligation.

In the original NAM, the primary outcome variable is the behaviour of interest. However, where we have placed duty of care in the hypothesised PCC model would suggest that the outcome from the NAM variables would be the behaviour-specific attitudes of the TPB. This may be an oversimplification and requires empirical testing. It may well have a unique influence on behaviour directly, bypassing the TPB variables. However, the place of duty of care within the PCC model should not affect the present goal of developing a scale to measure it.

We will now briefly define the key NAM variables as these will constitute the hypothesised *a priori* dimensions of the duty of care scale [7]. While activation is the first step in the model sequentially, we will begin with Personal Norms as they are the cognitive foundation of feelings of moral obligations and thus, central to the model.

*Obligation stage*. ***Personal norms*** are an individual’s beliefs or self-expectations about how they ought to behave in a particular situation. Differing from the more commonly used ‘social norms’ which are the expectations or standards of *others* for how we should behave, personal norms are our own *personal* expectations for behaviour. Of course, these self-expectations originate in social learning, but over time they are internalised and modified in each individual [8]. Personal norms also stem from values, but differ from them in that they are situation/behaviour-specific while values are trans-situational and more enduring (for more detail on how personal norms differ to and complement values, see section 3.1 of Schwartz and Howard [26, pg 238]).

Where social norms motivate behaviour through social sanctions, personal norms motivate behaviour through sanctions related to the self-concept and associated self-evaluative **emotions** [8, 10]. That is, a person may be motivated to behave in line with their personal norms to avoid negative self-evaluation and feelings of guilt, failure, or loss of self-esteem, or to gain or maintain feelings associated with positive self-evaluation like pride, security, or enhanced self-esteem. Consequently, these internalised norms are self-reinforcing, personal standards for behaviour [8] and as such, are particularly promising with regards to behaviour change and the sustainability of that change. Additionally, this feature of personal norms whereby actions are evaluated in terms of the self-concept and the moral implications for one’s sense of self is what differentiates them from other attitudinal constructs (such as those included in the Theory of Planned Behaviour), which make evaluations based on material, social, and psychological payoffs [26]. With regards to the basic propositions of the NAM, personal norms are the cognitive foundation for feelings of moral obligation (the primary motivator in the NAM). That is, feelings of moral obligation are the product of *activated* personal norms combined with anticipated self-evaluations/emotions [8]. Furthermore, from an operational perspective, it is difficult to measure the *feeling* of moral obligation that motivates behaviour in specific situations; it is more feasible to measure the salient beliefs these feelings derive from (i.e., personal norms) which are potentially more stable [8]. This is a similar approach to that commonly employed when operationalising the Theory of Planned Behaviour, measuring salient beliefs as opposed to attitudes directly.

*Activation stage*. ***Awareness of need*** and ***problem awareness*** are concerned with the *activation* of personal norms and have been used somewhat interchangeably in NAM studies. They refer to the extent to which a person is aware of the need of the focal entity, whether that be another person, an animal, the environment etc. While conceptually similar, in practice, awareness of need is more often used with regards to more specific needs (e.g., reducing car use to reduce climate change), while problem awareness has been used more generally (e.g., climate change as a major issue of concern). Additionally, it is worth noting that several studies, particularly within the environmental domain, have used the term “awareness of consequences” to describe awareness of need or problem awareness. However, in Schwartz’s original presentation of these concepts, awareness of consequences is described as a trait-like variable (described later), not a situational activator [20].

Schwartz [8] highlights that the relationship between awareness of need, personal norms, and behaviour can be influenced by several factors. Initially, to reach the threshold of basic awareness, the prominence and clarity of the need are important, as well as the individual’s personal receptivity to the need cues. In the case of dog care, receptivity to need cues would also include understanding/knowledge of what a need cue is e.g., understanding dog behaviour and body language that is indicative of a problem for the dog. Indeed, anecdotally, many dog owners are receptive to some need cues like problem behaviours (e.g., they notice that their dog is destroying furniture), but aren’t aware that they are a sign of compromised welfare or may misconstrue them (e.g., they consider the dog is being spiteful). Once the threshold of basic awareness of need is reached, the intensity or seriousness of the need then also has an impact on behaviour. That is, if we consider the behaviour of taking the dog to the vet, action is more likely to occur in response to more intense need (e.g., dog with broken leg) than less intense need (e.g., dog with skin condition). Stimulus overload is also likely to influence awareness of need by reducing attentiveness to need cues. That is, owners that have “a lot going on”, perhaps with work or with other family commitments, are likely to not be as attentive to the needs of their dog and miss some of these need cues. In these situations, the time available to interact with the dog and therefore time available to notice need cues would also be impacted. These examples highlight some of the wide range of potential factors that can influence an owner’s awareness of need or problem awareness.

***Efficacy*** and ***outcome efficacy*** are also terms used interchangeably in the literature. Once a need has been recognised efficacy refers to the perception/recognition/identification of potential actions that could relieve the need.

Following from efficacy, ***ability*** is the extent to which the person believes they can perform the identified actions to relieve the need. In this way it is similar to perceived behavioural control from the Theory of Planned Behaviour. This is a key area for neutralising obligations later in the process through denial of ability.

***Ascription of responsibility/situational responsibility*** is often described as the acceptance of responsibility for the consequences of performing or not performing the identified behaviour/s. However, Schwartz originally described it as “a sense of connection or relatedness with the person in need” and “the sense of some responsibility to become involved with the needy” [8, pg 246]. He also differentiated responsibility from obligation in that: “Feelings of obligation are directed toward the performance of specific acts; their strength is a function both of the connection with the needy (responsibility) and of the implications of the act for self-evaluation.” Existing role relationships (i.e., relationships with reciprocal role expectations of each party) automatically implicate a sense of responsibility and this is likely the case for many dog owners. However, there are several other sources of responsibility, the most relevant for dog ownership being the concept of dependency. As such, the greater the owner’s perception of the dog’s dependency on them, presumably the greater their ascribed responsibility. On the contrary, if an owner perceives their dog and its welfare to be relatively independent of the owner, their acceptance of responsibility is likely to be lower and perhaps even facilitate a denial of responsibility.

***Awareness of consequences*** has been perhaps the most commonly confused concept from the NAM, yet is also one of the most commonly used. In the original descriptions, awareness of consequences is described as a general predisposition or trait-like “tendency to become aware of the consequences of one’s behaviour for others” [8, pg 229] or the tendency to “perceive situations in terms of the consequences their own behaviour has for others” [8, pg 225]. It is trans-situational and as such, it facilitates, but is not the same as the situational awareness of need described above. It was originally measured using a general scale, not specific to the behaviour of interest [8, 20].

*Defence stage*. Where the costs of action outweigh the benefits, defence mechanisms may be employed to neutralise feelings of moral obligation. Each of the original situational activators are potential targets for defence against action. That is, one may deny the reality or severity of the need or problem, deny their ability to perform the actions required, or deny their responsibility in the particular situation.

In addition to the situational defences described above, ***responsibility denial*** has been identified as a trans-situational trait likely to influence this process. Like awareness of consequences, it is a general predisposition to deny responsibility for the consequences of one’s actions [8]. There is a 28 item set that is designed to measure this across settings [8].

*Care beliefs and standard of care*. While the NAM provides a good framework for the moral obligation component of duty of care, there is one other element to be considered: the level of care that is implied by the obligation. That is, different owners may have similar feelings of moral obligation to provide care for their dog, but the actual standard of care they perceive to be appropriate may differ. These differences could arise from differences in value-based judgments and differences in knowledge about the needs or capacities of dogs. For example, one owner may consider that an appropriate level of care for their dog is for it to sleep inside on a bed, while another may consider it more appropriate for dogs to sleep outside in a more “natural” state.

**Fig 4:**
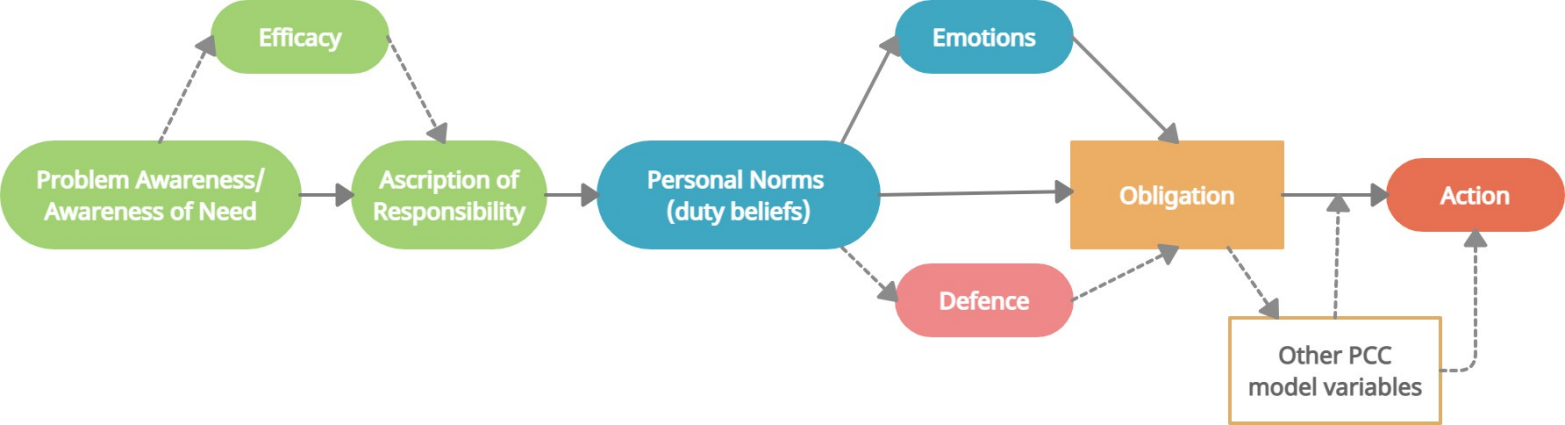
Hypothesised model of NAM elements for use in the duty of care scale. Relationships supported by previous research depicted by solid arrows. Postulated relationships in dashed lines.

*Model structure*. As mentioned above, different studies have used different statistical models of the NAM, typically either a moderation or a mediation model. A review by de Groot and Steg [22] of the use of the NAM in the context of environmental work, argued that the mediator model is more appropriate. However, this review only modelled three NAM variables (personal norms, awareness of consequences [awareness of need/problem awareness], and ascription of responsibility). The mediator model suggests that a person must be aware of the problem or need before feeling responsible for it [23]. Additionally, they tested a range of models to determine the appropriate place of emotions, finding that they best served as mediators between personal norms and intentions, but with personal norms also having a direct effect on intentions as well. Few studies have used efficacy, ability, and defence in their NAM models, so it is unknown how they should interact with the other elements. However, we consider it likely that they work in similar ways to emotions and as such, potentially have a mediating effect within the activation stage and the norm-obligation-action transition. Fig 4 provides a graphical depiction of the hypothesised model structure.

*Evaluation of existing instruments*: *Is a new scale needed*?. There are many instruments currently available for assessing various characteristics of human-animal interactions. Indeed, Wilson and Netting [27] identified 140 different tools that had been developed up until 2010. Since then, many more have been added to this list. However, there is presently no standardised, psychometrically validated scale to evaluate the various concepts related to duty of care as described here. Other studies have typically only used a small number of items (one-three) to measure obligation [18, 19]. However, given the proposed multidimensional nature of duty of care we consider that a more extensive scale is required.

#### Deductive item generation: Literature review and existing scales

Existing scales regarding the Human-Animal Relationship were examined for items relevant to the dimensions outlined in the domain identification. Additionally, studies that utilised the NAM in other contexts were drawn on to structure other items not previously used in the human-animal literature.

One important consideration was the level of behavioural specificity at which the items would be focussed. It is well recognised that the more specific the items, the better they are at predicting individual behaviours (i.e., single act criteria) [28]. However, when examining a general concept such as appropriate dog care, there are a multitude of individual behaviours that constitute this. One potential solution is to utilise the concept of a multiple act criterion [29]. That is, measuring general attitudes towards dog care is more likely to be predictive of a *set* of related behaviours in a single behavioural domain with the same target (e.g., the dog) [8]. By examining a range of specific actions, a class of behaviour is made which is more likely to be predicted by a general attitudinal antecedent. As such, in initial item generation, we included both items regarding general dog care and welfare as well as specific management behaviours to cover both single and multiple act criteria. The specific management behaviours used were those that were considered more discretionary (e.g., spending time, exercise, preventative health care) as they would likely have greater variability in related attitudes than foundational care behaviours (e.g., feeding, providing water).

#### Inductive item generation: Interviews

To elicit the “real-world” views of dog owners, semi-structured qualitative interviews were conducted with adult (18yr+) dog owners. Self-selection bias is a major challenge when studying pet owner attitudes as individuals who are more engaged and interested in dogs are more likely to volunteer for dog-related research. As such, we used several measures to reduce this bias and to capture a broader range of views. Recruitment was conducted in two ways. Firstly, posts were made in Facebook community groups, but these were limited to non-dog related groups e.g., “Buy, Swap, Sell” or Community Noticeboard pages. Secondly, the Royal Society for the Prevention of Cruelty to Animals (RSPCA) Victoria (Australia) assisted by emailing the study advertisement to people who had engaged with their “Latrobe Loves Pets” community outreach program. The Latrobe Loves Pets program provided support (e.g., assistance with veterinary bills, free pet food or supplies) to individuals who were facing challenges providing care for their animals. In doing so, we hoped to capture parts of the community that may face barriers to providing optimal pet care. An incentive of a $50 visa gift card was provided for each participant to both encourage participation by people who are not dog-enthusiasts and to compensate participants for their time. Additionally, we used a brief screening questionnaire in the Expression of Interest (EOI) form which focussed on beliefs about dogs and key management practices (see S1 File). This enabled us to select participants with varied views from a wider pool of EOIs. A total of 363 EOIs were received.

Thirteen (*n* = 13) interviews were conducted via video call (Zoom Video Communications Inc, San Jose, CA, USA) in September 2020, January 2021, and February 2021. Participants included 4 males and 9 females ranging in age from 25–54, living in various areas of Victoria, Australia including inner city Melbourne, suburban Melbourne, and regional/rural Victoria. Participants also varied in their household characteristics including families with young children, families with adult children (left home), couples, singles, and singles living in share housing. The dog-owning experience of participants also varied; some had extensive experience caring for dogs while others were first-time dog owners. Each interview lasted for approximately 1 hr and all interviews were conducted by the first author (CRG). To begin each session, a quick-response sentence completion exercise was conducted to elicit “top of mind” responses to key concepts and to facilitate further conversation. The remainder of the interview was semi-structured, addressing themes of general beliefs about animals and their own pets, general motivation for management activities, responsibility, perceived control/barriers, emotions, and finally, duty of care (full interview protocol available in S2 File). Duty of care was deliberately left until the end of the interview to see if it would be mentioned by participants unprompted earlier in the interview.

The Zoom session for each interview was recorded and fully transcribed for thematic analysis. Transcripts were manually coded using NVivo Version 12 (QSR International Pty Ltd. 2018) using a combination of inductive and deductive coding. While some themes/nodes derived from the domain identification and background theory, others were developed in response to the data. All nodes were reviewed, consolidated, and amended in an iterative process. Questionnaire items were then developed to reflect these key themes (e.g., I brought my dog into my household so I have a moral obligation to care for them). Additionally, some items were derived directly from statements made by interviewees (e.g., I owe my dog fun times).

#### Initial item pool

A full list of the generated items can be found in the (S3 File) with their relevant source. The majority of items were developed in a Likert style using a 7-point response scale from ‘strongly disagree’ to ‘strongly agree’.

For two of the dimensions (ascription of responsibility and problem awareness/awareness of need), two different approaches to their measurement were developed for testing and comparison. For ascription of responsibility, the first set of items were more general “agree-disagree” items (e.g., I feel completely responsible for my dog’s safety). The second set focussed on more specific states of the dog (i.e., the consequences of care and management) and asked participants “To what extent do you feel responsible for the following:”, then used piped text to insert the dog’s name into the individual items e.g., “[dog’s name] being happy”. The response scale was a 5-point scale from “not at all responsible” to “completely responsible”. For problem awareness/awareness of need, both approaches used the same response scale (agree-disagree) but the first set was more general in nature (e.g., My dog could be happier) while the second set pertained to the specific management or interactive behaviours (e.g., If my dog does not get enough exercise their welfare will suffer).

The three items developed for efficacy could also be interpreted as ability (or perceived behavioural control). That is, in saying “There are things I could do to improve my dog’s life” it includes both the identification of behaviours required to improve the dog’s life and a judgement of whether the person could do them. As such, we combined these elements of the NAM for the present scale. Importantly, when used in the context of the PCC model, additional items would be included to assess perceived behavioural control under the Theory of Planned Behaviour section, and general knowledge, which would also add to this. Hence the few items (three) used here are justified.

### Stage 2: Item reduction

#### Online survey

This stage of scale development involves delivering the full set of generated items (S3 File) to a large sample of the target audience to facilitate factor analysis and item reduction. To do so, an online survey was constructed using Qualtrics software, Version April 2021 (Qualtrics, Provo, Utah, USA). The survey comprised 3 main sections and 8 subsections based on the dimensions identified in domain identification.

1. Demographics (to ensure adequate representation of different demographics)
2. Duty of Care scale items (see S3 File)
  - Personal norms (duty beliefs)
  - Ascription of responsibility
  - Problem awareness and awareness of need
  - Efficacy/Ability
  - Emotions
  - Standard of care
3. Existing scales (for testing construct validity)
  - Lexington Attachment to Pets Scale
  - Monash Dog Owner Relationship Scale

Demographic questions included gender, age, locality (urban, suburban, regional), number of dogs in the household, dog’s name, source of dog acquisition, and age at acquisition. The dog’s name was recorded as this was used in later questions to elicit a more contextualised response than simply referring to “your dog” (e.g., “to what extent do you feel responsible for [Dog’s name]’s mental state”).

At the end of each page (subsection) there was an optional text box for participants to provide feedback on the items (e.g., comprehension and clarity) or to indicate if there was some aspect of that concept that had not been covered by the items.

The Lexington Attachment to Pets Scale (LAPS) [30] and Monash Dog Owner Relationship Scale (MDORS) [31] are both existing, psychometrically validated scales and were included in the survey to investigate construct validity (see Stage 3: scale evaluation).

The survey was disseminated through social media, predominantly Facebook and Twitter. As with the interview recruitment, posts were made to primarily non-dog related Facebook community groups e.g., ‘Buy, Swap, Sell’ or ‘Community Noticeboard’ pages. Data collection occurred from June 8, 2021 to July 20, 2021.

#### Exploratory factor analysis

Each subsection of the duty of care scale was analysed separately. This began with examining descriptive statistics for each item, inter-item correlations, and the qualitative feedback on the items (provided in the text boxes at the end of each section) to determine which items should be included in the subsequent exploratory factor analysis (EFA). Items with low inter-item correlations (<|0.3|) [7] and substantial negative qualitative feedback were excluded from factor analysis. Where possible, only items that did not demonstrate significant skew (skewness <|1|) were included [32]. However, for some subscales (e.g., duty beliefs), this was not possible as most items in the subscale demonstrated skew. As such, the principal axis factoring (PAF) extraction method was used for the EFA as it is the most robust method for skewed variables [33]. Factors were extracted based on both eigenvalues and the scree plot. Small coefficients (absolute value less than 0.33) were suppressed [34, p. 649] and variables that did not load were removed before reanalysing the smaller set of items. Rotation was not required as all but one analysis yielded a single factor solution.

After initial EFA solutions were found, the internal consistency of each factor was tested using Cronbach’s alpha (α). If any items could be removed to improve internal consistency they would have been, however this was not the case for any of the scales. Factor scores were saved using the regression method and descriptive statistics were conducted to examine their distribution. These analyses were performed with IBM SPSS Statistics for Windows, version 27 (IBM Corp., Armonk, NY, USA).

### Stage 3: Scale evaluation

The final stage of scale development involves various evaluations of the scale’s dimensionality, reliability, and validity. While not all recommended evaluations were able to be included in the present study for practical reasons (see discussion), we conducted structural equation modelling, calculation of simple scale scores, and construct validity assessment.

#### Structural equation modelling

Following the EFA, zero-order correlations were conducted on the factor scores to provide a preliminary understanding of the relationships between the different factors. Subsequently, structural equation modelling (SEM), combining both confirmatory factor analysis (CFA) and path analysis, was conducted to examine the structure and fit of the full model. SEM was performed using IBM SPSS Amos for Windows, version 27.0.0 (Amos Development Corp., Wexford, PA, USA). While the PAF extraction technique was used for the original EFA as it is most appropriate for skewed variables, maximum likelihood (ML) is the only method available in Amos for SEM. As such, to ensure this was appropriate for the SEM we reanalysed the original data with the maximum likelihood method which produced very similar results to the PAF method. The hypothesised model (Fig 4) was used as a basis for the SEM with modification indices inspected to determine if improvements in model fit could be achieved.

#### Scale scores

Once the structure and items were confirmed, simple scale scores were calculated for each subscale by summing the values for each relevant item. These were then compared to the factor scores using correlations. This is an important step to simplify future use of the scale, assessing the validity of using simple scale scores as opposed to factor scores.

#### Construct validity

The relationships between the duty of care constructs and the LAPS and MDORS scales were assessed for construct validity. The concepts of attachment to pets (LAPS) and the dog-owner relationship (MDORS) were expected to correlate with the duty of care constructs, yet remain distinct. In this way, these scales were used to assess both convergent and discriminant validity.

The MDORS and LAPS each have three validated sub-scales. Scores for each subscale were calculated by summing their relevant items. Additionally, an overall score was calculated for each scale by summing all items. Zero-order correlations were conducted between the MDORS and LAPS scores and the duty of care simple scale scores.

## Results

### Participant demographics

A total of 538 individuals participated in the online survey (455 complete, 83 incomplete) of which 82.5% were female, 16.5% male, and 0.9% non-binary. Participants ranged in age from 18–86 with a mean age of 44.90 (*SD* = 14.73) and age was normally distributed (skewness = 0.31, kurtosis = -0.77). Most participants had 1 (59.2%) or 2 (29.4%) dogs in their household with the majority acquiring them from a breeder (42.8%) or rescue situation (13.9% animal shelter, 14.3% rescue group). Most dogs were acquired at a young age, with 75.2% (cumulative) acquired before 1yr old, 57.6% by 12 weeks and 36.1% by 8 weeks.

### Exploratory factor analysis

Of the six proposed subscales, four produced psychometrically satisfactory results (personal norms, ascription of responsibility, awareness of need, and efficacy). The key metrics for each EFA solution are provided in Table 1.

**Table 1:** Key diagnostics of exploratory factor analyses for each sub-section of the duty of care scale that produced satisfactory solutions.

	Personal norms (duty beliefs)	Ascription of Responsibility	Awareness of Need	Efficacy
**Number of Factors**	1	1	2	1
**Total variance explained**	35.83	47.44	43.58	59.96
**Reproduced correlations nonredundant residuals**	3 (14%)	11 (24%)	11 (24%)	0
**KMO measure of sampling adequacy**	.86	.92	.75	.67
**Bartlett’s test of sphericity**	< .001	< .001	< .001	< .001
**Determinant**	.203	.008	.045	.336
**Reliability (Cronbach’s alpha)**	.78 (*n* = 7)	.87 (*n* = 10)	Factor 1: .84 (*n* = 4)	.80 (*n* = 3)
			Factor 2: .73 (*n* = 6)	

#### Personal norms (duty beliefs)

All items were significantly skewed with the exception of “I feel obligated to provide my dog with as close to a ‘natural life’ as possible”. However, there were several comments in the feedback about this item being confusing. Therefore, it is likely that the greater spread for this item is more reflective of variation in interpretation of the question as opposed to actual differences in underlying beliefs. As such, this item was removed prior to factor analysis. Additionally, there were many comments objecting to the use of the phrasing to “owe” dogs, so all items using this term were removed. A single factor solution comprising 7 items was found, accounting for 35.8% of the total variance and with an acceptable level of internal consistency (α = .78) (Table 2). Factor scores remained significantly skewed (skew = -1.82, *SE* = 0.11).

**Table 2:** Item loadings for exploratory factor analysis of personal norms (duty beliefs) dimension of the duty of care subscale.

	Factor
	1
DB_2 I feel a strong personal obligation to ensure my dog is happy	.700
DB_3 I feel a strong personal obligation to ensure my dog is healthy	.673
DB_10 If you bring an animal into your home, it is your duty to make sure they are happy	.642
DB_12 In the same way that people have basic rights, like food, medical care, and housing, so too do dogs.	.585
DB_16 My dog trusts me, so I must live up to that trust	.561
DB_7 My obligations to my dog stem from them being a part of the family	.499
DB_19 We don’t have any particular obligations or duties to our dogs	-.496

*n* = 455.

Extraction Method: Principal Axis Factoring. 5 iterations required.

Given that the resulting factor scores remained skewed, we considered rewording the items to use more moderate language, making the items less likely to elicit “strongly agree” responses. Reworded items were trialled on a new group of participants as part of a separate study (*n* = 88). All reworded items were skewed except two items (all reworded items available in S4 File). The least skewed item was “My duty to care for my dog is. . .” with a 7-point response scale from “the least important thing in my life” to "the most important thing in my life” (skew = -.58, *SE* = .26). The second asked "How personally obligated do you feel to minimise your dog’s negative experiences in life?” with a 7-point response scale from “Not obligated at all” to “Completely obligated” (skew = -.88, *SE* = .26). Factor analysis and SEM could not be performed on this sample as there was not a sufficient sample size.

#### Ascription of responsibility

The first approach to measuring ascription of responsibility (general; agree-disagree) demonstrated less variability, lower inter-item correlations, and more skew than the second approach (specific states; “How responsible would you feel…”). Many of the individual items used for the second approach were normally distributed. Highly skewed items were removed before all remaining items from both sets were pooled together for the EFA. A 10-item, single factor solution was found accounting for 47.44% of the variance with a good level of internal consistency (α = .87) (Table 3). Despite the low loading of item AR1_14 (-.332), we considered it important to retain this due to its unique semantic content, reflecting an element of denial of responsibility. Interestingly, the most skewed items (that were removed prior to factor analysis) often related to positive states (e.g., level of responsibility felt for dog being happy, healthy, or safe).

**Table 3:** Item loadings for exploratory factor analysis of “ascription of responsibility” dimension of the duty of care subscale.

	Factor 1
AR2_16 If [dog’s name] felt insecure or afraid	.809
AR2_10 If [dog’s name] was anxious	.798
AR2_9 If [dog’s name] was depressed	.775
AR2_1 [dog’s name] ‘s mental state	.768
AR2_8 [dog’s name] being free of fear	.714
AR2_18 If [dog’s name] was lonely	.694
AR2_15 If [dog’s name] engaged in problem behaviours like destroying things or excessive barking	.649
AR2_17 If [dog’s name] was bored	.648
AR2_19 If [dog’s name] was aggressive to other people or dogs	.568
AR1_14 During challenging times like financial hardship, changes in personal circumstances, or when time is limiting, it is reasonable to lower our expectations for dog care and welfare.	-.332

*n* = 462.

Extraction Method: Principal Axis Factoring. 4 iterations required.

#### Problem awareness and awareness of need

In contrast to the ascription of responsibility items, the results of the general set were less skewed than those of the specific set. After removing items based on skew and inter-item correlations, both sets were pooled together for EFA. After an initial two factor solution was found, only the top six items loading on the second factor were retained to ensure a similar number of items for this section as the previous. This final 10-item, two factor solution accounted for 43.58% of the variance (Table 4). Factor 1 reflects the concept of “problem awareness” (PA) and was named as such. Factor 2 reflects an awareness of the consequences or impact of management behaviours on the dog’s behaviour and welfare. However, to avoid confusion with the broader trait-like variable “awareness of consequences” (see domain identification section), we will call this “awareness of impact” (AoI). Both factors exhibit acceptable-good internal reliability (α_PA_ = .84, α_AoI_ = .73).

**Table 4:** Item loadings for exploratory factor analysis of “awareness of need” dimension of the duty of care scale.

	Factor	
	1: Problem Awareness	2: Awareness of Impact
AN.Gen_8 My dog could lead a better life than they currently lead	.824	
AN.Gen_6 My dog could be happier	.789	
AN.Gen_5 My dog’s welfare could be better	.759	
AN.Gen_7 My dog could be healthier	.635	
AN.Spec_5 If my dog does not get enough mental stimulation their welfare will suffer		.725
AN.Gen_12 How I manage my dog affects his/her behaviour		.608
AN.Spec_2 Providing toys/puzzles/enrichment items helps keep dogs occupied and not engaging in problem behaviours		.555
AN.Spec_4 If my dog does not get enough exercise their welfare will suffer		.538
AN.Gen_1 Providing good care for my dog improves their behaviour		.520
AN.Gen_2 Problem behaviours in dogs (e.g. destructive behaviours, excessive barking) are often the result of their needs not being met		.481

*N* = 462.

Extraction Method: Principal Axis Factoring. 7 iterations required.

#### Efficacy/Ability

These items were not significantly skewed, displayed a good spread of responses, and had good inter-item correlations. A single factor solution accounting for 59.96% of the variance was found with an internal consistency of α = .80 (Table 5).

**Table 5:** Item loadings for exploratory factor analysis of “efficacy/ability” dimension of the duty of care scale.

	Factor
	1
Efficacy_3 There are things I could do to improve my dog’s life	.891
Efficacy_1 There are things I could do to make my dog happier	.810
Efficacy_2 There are things I could do to make my dog healthier	.590

*N* = 486.

Extraction Method: Principal Axis Factoring. 5 iterations required.

#### Emotions

All items demonstrated good inter-item correlations and there was no negative qualitative feedback about any of the items. However, all items except two were highly skewed with little variation. Initially EFA was trialled with all items. A 3-factor solution was found to be the best fit for these data. However, when these factors were incorporated into the SEM there were issues with overly large regression weights (β>1). Consequently, we attempted to re-do the EFA excluding the most skewed items (skew>2). A reasonable two factor solution was found, however the distribution of the resultant factor scores for factor 1 were still heavily skewed (skew = -2.28) and the reliability was unacceptable (α = .66). Factor 2, despite consisting of only two items, demonstrated better reliability (α = .76) and was not skewed (skew = -.16). However, when trialled in the SEM, we found that it had no relationship with the duty beliefs (β = 0.01). As such, it was concluded that the items developed for assessing the emotional component of the NAM were inadequate and excluded from the model.

While the qualitative feedback did not highlight any issues with the items themselves, several participants felt the need to explain that they are more motivated by positive emotions (e.g., happy, rewarding) than by negative emotions (e.g., guilt, shame).

#### Care beliefs/standard of care

Similarly to the emotions section, all items except two were highly skewed with little variation. A similar process to that described for emotions was conducted with similar results. The initial factor solution caused problems in the SEM and the revised EFA (excluding the most heavily skewed items) failed to produce acceptable factors. As such, this subsection was excluded from further analyses and SEM.

### Full scale evaluation: Inter-factor correlations and structural equation modelling

Following the EFA a total of 30 items remained. The inter-factor correlations demonstrated good relationships between the subscales in line with the hypothesised model (Table 6).

**Table 6:** Zero-order correlations between duty of care subscale factor scores.

	Duty Beliefs	Ascription of Responsibility	Problem Awareness	Awareness of Impact	Efficacy
Duty Beliefs	1				
Ascription of Responsibility	.439**	1			
Problem Awareness	-.192**	-.204**	1		
Awareness of Impact	.349**	.480**	.006	1	
Efficacy	-.027	-.081	.748**	.126**	1

*n* = 455, **p < .01.

**Fig 5:**
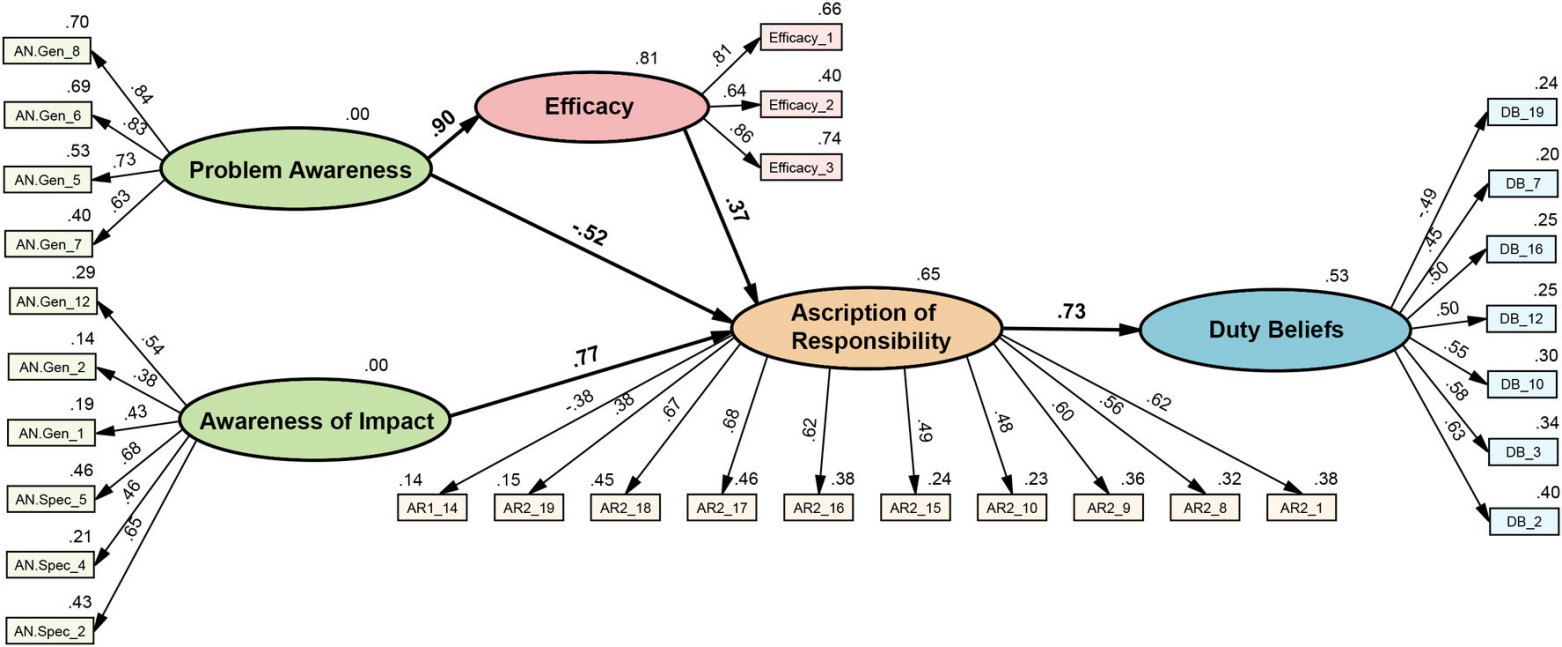
Structural equation model of the duty of care scale. *n* = 455, Chi-square = 640.25, *df* = 351, item labels defined in Tables 2–5.

All items were entered into the SEM according to the hypothesised model structure (Fig 4). Overall, the model demonstrated excellent relationships between model elements (Fig 5) and good model fit: PCMIN/DF = 1.824, CFI = .947, RMSEA = .043, SRMR = .0458. Additionally, the variance accounted for in each of the endogenous (dependent) variables within the model was very good: *R*^2^_efficacy_ = .81, *R*^2^_AoR_ = .65, *R*^2^_dutybeliefs_ = .53. Two relationships between latent variables warrant highlighting. Firstly, problem awareness had a negative relationship with ascription of responsibility; that is, the more a person considered that their dog’s life could be better, the less responsible they felt for a range of negative states. Secondly, efficacy functioned as a suppressor variable, increasing the strength of the relationship between problem awareness and ascription of responsibility from -.2 to -.52.

### Simple scale scores

Correlations between the subscale factor scores and simple scale scores (summed items) were excellent: *r*_duty_ = .895, *r*_responsibility_ = .943, *r*_problem_ = .986, *r*_impact_ = .968, *r*_efficacy_ = .970; all *p* < .001, *n* = 455. This scale is not designed to be used as a unidimensional scale and as such, an overall total was not calculated or examined.

### Construct validity

Correlations between the duty of care subscales and the MDORS subscales were primarily weak-moderate, while the relationships with LAPS subscales varied from weak to strong (Table 7).

**Table 7:** Zero-order correlations between duty of care subscale factor scores and Monash Dog Owner Relationship Scale (MDORS) and Lexington Attachment to Pets (LAPS) total scale scores and subscale scores.

	Duty Beliefs (personal norms)	Ascription of responsibility	Efficacy	Problem Awareness	Awareness of Impact
MDORS total	.265**	.177**	.083	.033	.323**
Dog-Owner Interaction	.310**	.267**	-.015	-.101*	.354**
Perceived emotional closeness	.393**	.352**	-.017	-.109*	.345**
Perceived costs	-.257**	-.333**	-.191**	-.285**	.131**
LAPS total	.590**	.477**	-.002	-.142**	.401**
General attachment	.585**	.474**	-.072	-.227**	.411**
People substituting	.468**	.390**	.039	-.077	.327**
Animal rights/welfare	.527**	.400**	.029	-.056	.315**

*n* = 455 *p < .05, **p < .01.

## Discussion

This research aimed to gain a better understanding of duty of care in companion dog owners and develop a novel, multi-dimensional scale to assess it. Drawing on both psychological theory and real-world views of dog owners, we have developed a 30 item scale with 5 dimensions (see S5 File for final scale). Each dimension represents a unique aspect of duty of care based on Schwartz’s Norm Activation Model; duty beliefs, problem awareness, awareness of impact, efficacy, and ascription of responsibility. All items use either a 5- or 7-point response scale and subscale scores can be easily calculated by summing the relevant items. Given the distinct nature of the different subscales, future work could use all subscales or a select few, depending on the research aims. However, the subscales are not intended to be summed together for an overall “duty of care score”.

Subscale reliability and validity demonstrated good results. The subscales have good internal consistency as measured by Cronbach’s alpha. The significant correlations between the duty of care subscales and the Monash Dog Owner Relationship Scale and the Lexington Attachment to Pets Scale support their convergent validity. However, the strength of the relationships (ranging from weak to strong), indicate that they are not measuring identical underlying concepts and therefore, are not redundant. Test-retest reliability was not assessed in this study. Additional work is also required to assess the scale’s predictive validity i.e., the extent to which the scale predicts owner behaviour and dog welfare outcomes. This will be a significant undertaking as it will need to be assessed in the context of the full Pet Care Competency model (Fig 1) and involve assessment of both owner behaviour and dog welfare outcomes.

In addition to simply developing a measurement tool, this process has provided insights into the nature of duty of care. To elaborate on this, each of the subscales will now be examined in turn.

The **duty beliefs** subscale reflects Schwartz’s central concept of ‘personal norms’, i.e., an individual’s beliefs about how they ought to behave in a particular situation. Despite the subscale scores being significantly skewed, thereby reducing the statistical ability to detect significant relationships with other variables, good relationships with the other duty of care subscales were still found. This skew in duty beliefs could be the result of two factors. Firstly, the sample of dog owners recruited may have overrepresented those that have strong duty beliefs and failed to capture those with weak duty beliefs. Secondly, the items themselves may not be sensitive enough to differentiate between more nuanced levels of duty beliefs. We consider that the skew is likely a combination of both of these factors. The fact that our attempts to reword the items also yielded highly skewed items suggests that the type of dog owners recruited primarily through social media and those that readily participate in research, generally have strong duty beliefs. Such self-selection bias is well recognised in research and difficult to overcome [31, 35, 36]. Now that the number of items is greatly reduced, the next stages of scale validation would benefit from more targeted recruitment, potentially including groups that are likely to have weaker duty beliefs. This could include individuals who have relinquished their dog or been investigated for neglect or cruelty. Skewed variables typically reduce the ability of correlation or regression-based analyses (like SEM) to detect significant effects. However, the relationships in the SEM and construct validity correlations were still statistically significant with good effect sizes. As such, while demonstrating less than optimal psychometric properties, we consider the duty beliefs subscale to still be adequate in the context of the model. Future work could also incorporate the two reworded items that demonstrated less skew and assess their fit within the subscale and broader model.

This tendency for high levels of duty beliefs within a sample recruited from the general public may indicate that duty beliefs are not major predictors of welfare problems. That is, if the majority of dog owners have very high levels of duty beliefs, yet behavioural and welfare problems are highly prevalent within the general population [1, 37–39], perhaps they are not a result of a lack of duty beliefs, but rather the other situational or activation factors in the model such as problem awareness or taking responsibility for negative outcomes (ascription of responsibility). There is some evidence of dog owners having limited ability in interpreting dog body language or recognising less obvious signs of stress [40–42]. This would likely be a contributing factor to an inability to detect when there is something wrong or when there is a negative impact of their management. This requires further research that simultaneously evaluates dog welfare outcomes and the various duty of care elements, but it is an important line of enquiry for the identification of potential intervention targets.

The items included in the final solution for **ascription of responsibility** reflect the owner’s feelings of responsibility for negative affective states and problem dog behaviours. This is consistent with how ascription of responsibility is often used in the NAM literature, although not fully consistent with Schwartz’s original conceptualisation as outlined in our domain identification. Items that reflected the dependency of dogs on their owners (consistent with Schwartz’s definition) such as “Dogs depend on their owners for everything they need to be happy and healthy” and “My dog is reliant on me to lead a good life”, were too heavily skewed to be considered psychometrically valid i.e., most people strongly agreed. This may be because this dependency is considered “a given” to the average dog owner and it is the more detailed level of feeling responsible for affective states such as their dog being anxious or depressed that produces variability in responses. Additionally, the most highly skewed of the more specific items (and consequently those excluded from the factor analysis) were those referring to positive states such as the dog being happy, healthy, or safe. This could suggest that dog owners tend to take more responsibility for positive states and less responsibility for negative states. This proposition will be considered further below with regards to some of the other relationships in the model. Though it is important to recognise the exclusion of positive states from the final scale and the implications this has for the model as a whole. Essentially, it positions the duty of care scale to be specifically focussed on welfare risks. While this is consistent with the general concepts of the NAM, we recognise that this does not reflect the general shift in thinking within animal welfare science to not only focus on negative states, but also the promotion of positive states and general wellbeing [43]. We do not consider this to be a problem for the scale itself, but simply something that users of the scale should take into consideration.

The **problem awareness** subscale reflects the owner’s perception or awareness of whether their dog could be happier, healthier, or lead a better life. This is predicated on the fact that given the high prevalence of dog welfare problems, the lives of most dogs could be improved to some extent. The interesting finding here is the negative relationship between problem awareness and ascription of responsibility. That is, the more an owner considers there to be a problem with their dog’s welfare (i.e., they could be happier or healthier), the less responsibility they feel for negative states the dog experiences. This seems to add to the previous observation regarding the skew of positive responsibility items, potentially reflecting a tendency towards taking responsibility for the good, but a denial of responsibility for the bad. While this could be a denial mechanism to alleviate guilt or negative self-assessment, it could also simply be a product of extenuating circumstances or perceived control. In support of the latter explanation, is that in parallel and independent of this negative relationship is a positive relationship between **efficacy** and ascription of responsibility. That is, the more a person considers there are things they could do to improve their dog’s life, the *more* responsibility they feel for their dog’s negative states. Finally, the very strong positive relationship between problem awareness and efficacy suggests that there is significant overlap between these two concepts; the more one considers their dog’s life could be improved, the more they consider there are things they could do to improve it. However, the opposite relationships with ascription of responsibility (positive vs negative) highlight that they are in fact distinct, despite their commonalities. There are a range of potential explanations for this. Take for example, a dog that has been adopted as an adult that had poor socialisation and training in its previous household and now demonstrates reactivity towards other dogs. The owner could be highly aware of the problem and recognise that there are things they can do to improve their dog’s life. Consequently, they do feel responsible for negative states to some extent, but at the same time, they do not feel fully responsible because “the damage was done” prior to them adopting the dog. Additionally, there is a substantive difference between being able to do things to improve your dog’s life and being able to prevent or alleviate negative states. That is, owners may recognise that they can easily play with their dog or feed them a treat to make them happy, but preventing or improving negative states may be much harder and perceived to be more out of their control, therefore they do not feel as responsible for them. So while owners may be cognisant of things they can do to improve their dog’s life, these are not necessarily the same thing as preventing negative states. That efficacy acted as a suppressor variable, accounting for some of the shared variance to allow the main effect between problem awareness and ascription of responsibility to be improved, further supports this interpretation.

There is then also the question of whether problem awareness reflects whether there is an actual problem or just the attentiveness of the owner. To truly determine this, future work would need to simultaneously assess the dog’s welfare to determine the accuracy of owner perceptions. However, given the negative relationships between problem awareness and all three MDORS subscales and the LAPS total and attachment scores, this could suggest that it could be reflecting actual welfare problems. This is because if it was that owners who are more perceptive, knowledgeable, or more “in tune” with their dog scored higher on problem awareness because they are simply more aware, we would expect to see a positive relationship between problem awareness and attachment or interaction. That is, the more attachment, interaction, and emotional closeness, the higher they would score on problem awareness. However, the data reflects the opposite. As such, the lower attachment etc. could either be a result of or contributing to the welfare problem, or a combination of both.

The **awareness of impact** subscale primarily represents the owner’s recognition of the impact of general management and their own behaviour on their dog’s welfare and behaviour. In some ways this is similar to Ajzen and Fishbein’s “attitude towards the behaviour” element of the Theory of Planned Behaviour (and its predecessor the Theory of Reasoned Action), which is a function of the person’s beliefs about the consequences of the behaviour in question [6]. However, as mentioned in the introduction, the Theory of Planned Behaviour is typically more specific with regards to individual behaviours than the present scale. Many studies have combined elements of the Norm Activation Model and the Theory of Planned Behaviour to improve the predictive validity of each e.g., [9, 44–47]. It will be interesting to see in future studies how these two models work together in the context of the full Pet Care Competency model to predict owner behaviour and dog welfare outcomes (Fig 1).

Although **emotions** and **standard of care** beliefs are likely important elements in the full context of the Pet Care Competency model, this study was not able to develop satisfactory methods of assessing them. Further qualitative work is required to better understand the range of dog owner views on these aspects in order to develop more sensitive measurement instruments.

The primary limitations of this work have been discussed in detail above. To reiterate, these include probable sampling bias towards highly engaged owners, that predictive validity is yet to be tested, and the reduced subscale sensitivity for duty beliefs, emotions, and standard of care. Additionally, as the work was conducted only in Australia and in English, further research is required to determine the generalisability of the scale to an international audience. Studies of test-retest reliability and confirmation of the model structure in a separate cohort would also be advantageous.

In all, the present study has made significant progress in understanding duty of care in companion dog owners and developing a standardised tool to assess the various factors that contribute to it. It has provided important insights into the nature of the different duty of care elements and their interrelationships. Additional work is now required to understand the predictive validity of the scale and the relative influence of its different dimensions on owner behaviour and dog welfare outcomes within the framework of the Pet Care Competency model. In doing so, we will be able to better identify appropriate targets for intervention programs aimed at improving owner behaviour and consequently, dog welfare.

## Supporting information

S1 File EOI screening questionnaire. (PDF)

S2 File Interview protocol. (PDF)

S3 File Full list of generated items with source. (PDF)

S4 File Reworded duty belief items. (PDF)

S5 File Final duty of care scale. (PDF)
