# Supplementary material for: Duty of care in companion dog owners: Preliminary scale development and empirical exploration
Source: PLoS One. 2023 May 17;18(5):e0285278. doi: 10.1371/journal.pone.0285278 (PMC10191305; doi:10.1371/journal.pone.0285278)
Supplement: S1 File — (PDF) [file pone.0285278.s001.pdf]

S1 EOI screening questionnaire

	Strongly Disagree	Disagree	Somewhat disagree	Neither agree or disagree	Somewhat agree	Agree	Strongly Agree
1. Walking a dog is not necessary- having a backyard is sufficient	○	○	○	○	○	○	○
2. Dogs should be kept outside	○	○	○	○	○	○	○
3. It is ok to hit or kick a dog to teach it a lesson	○	○	○	○	○	○	○
4. Dogs are a bit overrated	○	○	○	○	○	○	○
5. I don’t pay that much attention to the dog really	○	○	○	○	○	○	○
6. Dogs don’t need attention	○	○	○	○	○	○	○
7. Dogs are more trouble than they’re worth	○	○	○	○	○	○	○
8. Dogs don’t need toys	○	○	○	○	○	○	○
9. It is important that dogs know their place in the household	○	○	○	○	○	○	○
10. We owe it to dogs to ensure they lead a good life	○	○	○	○	○	○	○
