# Supplementary material for: Duty of care in companion dog owners: Preliminary scale development and empirical exploration
Source: PLoS One. 2023 May 17;18(5):e0285278. doi: 10.1371/journal.pone.0285278 (PMC10191305; doi:10.1371/journal.pone.0285278)
Supplement: S2 File — (PDF) [file pone.0285278.s002.pdf]

S2 Interview Protocol

1. Welcome and give brief overview of project and purpose of the interview
    - Wanting to understand what members of the community think about animals
2. Recording and confidentiality
    - Remind the participant that the session will be recorded, but individual responses are confidential and will not be shared with anyone or any organisation outside of the research team.
    - Recording will start when we start the questions
3. Plain language statement/consent form
    - Ask if the participant has any questions
    - Ensure participant has read and signed the PLS

START RECORDING

1. Stems exercise

- To get us started, I am going to read out the start of a sentence and I just want you to finish that sentence with the first thing that comes to mind. There are no right or wrong answers, I just want to know the first thing that pops into your head.

**'Thinking about all animals in general…'**
I think animals are …
Animals make me feel …
I owe animals…
**'Thinking about all dogs in general…'**
I think dogs are …
Dogs make me feel …
I owe dogs…
**'Now thinking specifically about your dog/s…'**
I think [dog's name] is …
[Dog's name] makes me feel …
I owe my [dog's name]…

2. General Interview questions

**Research Topic (for reference only- not spoken)**	**Principle Question**	**Probing questions (if required)**
Basic beliefs about companion animals	Can you elaborate on some of the quick answers you just gave?	In general, what do you think about pets like cats and dogs?<br>Do you like having them?<br>What is their role or place?

Beliefs about their pet	Can you tell me a bit about [name of animal]?	Why did you get him/her?<br>Do you have any problems with them or their behaviour?<br>Do you enjoy spending time with them?<br>What about their behaviour, personality, intelligence (or lack of)?<br>Best qualities? Worst qualities?
General Motivation	What motivates you on a daily basis to look after your dog in the way that you do? To do those everyday tasks that may not be fun or enjoyable?	Do you feel that others expect it?<br>Do you feel that it's the right thing to do?<br>Do you do it because you feel guilty if you don't?
Responsibility	What sort of responsibilities do you have regarding [name of animal]?	What are you responsible for?<br>Are you responsible for their health?<br>What about their happiness?<br>At what point is it out of your hands and not your responsibility?
Duty beliefs	Do you think we have any particular duties or obligations to our pets?<br>What do you think we owe our animals?<br>What sort of standard of care do we need to provide?	If yes, what are they?<br>Why is that?<br>What actions are required to meet our obligations?<br>Where do you think these obligations come from? Are they a personal thing, something society expects, a legal thing?<br>How do our duties to pets relate to or differ from other animals like livestock or wild animals? What about other people or our children?<br>Where do animals fit in your priorities? Are there more important things that get in the way of looking after them?
Perceived Control	Are there any barriers to carrying out some of those obligations or duties?	Is it ever difficult to do some of these things?<br>Why is that?<br>How much control do you have over those barriers?
Emotions	How would you feel if you didn't carry out those obligations?	How would you feel if you didn't walk your dog?<br>Have you ever felt guilty about something you've done or not done with a pet?<br>What about not getting it vet care when it needed it or punishing it?<br>Do you think avoiding negative emotions motivates you in some way?

		Are there any other emotions that you think are relevant when thinking about how you care for your dog?
Duty of Care	What does the term 'Duty of Care' mean to you?	Have you heard of that before?<br>What do you think it means?<br>What sort of feeling does that term give you- a good one, a bad one?<br>Is it applicable to animals?

3. Debrief

Thank you very much for your time. The information you have provided has been very helpful. Now that we have finished, I just wanted to check-in with how you are feeling. Has this interview caused you any stress that you might need to talk to someone about?

If Yes:

- *Do you have a regular professional that you can talk to about this?*
  - If they do not have a regular professional, provide contact details for Lifeline: call 13 11 14 or SMS 0477 13 11 14.

4. Wrap Up

- Thank you for your time
- Would you be willing to be involved in the future (e.g. completing a survey)
- Any questions for me?
