# Supplementary material for: Duty of care in companion dog owners: Preliminary scale development and empirical exploration
Source: PLoS One. 2023 May 17;18(5):e0285278. doi: 10.1371/journal.pone.0285278 (PMC10191305; doi:10.1371/journal.pone.0285278)
Supplement: S3 File — (PDF) [file pone.0285278.s003.pdf]

S3 Full list of generated items with relevant source

Where a specific source has not been indicated, the item was developed by the researchers based on general application of the underlying concept to the context of dog care and welfare.

1. **Personal Norms (duty beliefs):**

General

Item	Source (if specific)
I owe my dog a good life	Interviews
I feel a strong personal obligation to ensure my dog is happy	
I feel a strong personal obligation to ensure my dog is healthy	
I am only obligated to make sure my dog has the basics to survive e.g. food, water, shelter.	Interviews (opposite)
I don't feel any obligation towards my dog	
I brought my dog into my household so I have a moral obligation to care for them	Interviews
My obligations to my dog stem from them being a part of the family	Interviews
Dogs can't look after themselves so we have a duty to look after them	Interviews
Dogs do so much for us so we owe them a good life in return	Interviews
If you bring an animal into your home, it is your duty to make sure they are happy and healthy	Interviews
Our duties to dogs arise from them being sentient, living creatures.	
In the same way that people have basic rights, like shelter and warmth, food, medical care, housing, so to do dogs.	Interviews
I feel a strong sense of duty towards my dog and their wellbeing	
I don't owe my dog anything	
It is a sign of a good person to care for your dog	(Rasool et al., 2019)
My dog trusts me, so I must live up to that trust	(Benz-Schwarzburg et al., 2020, Hens, 2009)
I owe it to my dog to provide them with the best care that I can	
Regardless of what other people do, because of my own values/principles I feel an personal obligation to provide a high standard of care for my dog	(Bamberg et al., 2007)
We don't have any particular obligations or duties to our dogs	(Glanville, 2017)
We are not obligated to facilitate positive experiences for dogs	(Glanville, 2017)
We are only obligated to reduce negative experiences for our dogs (e.g. hunger, pain, discomfort)	(Glanville, 2017)
We have a similar obligations to our dogs as we do to our children	Interviews
My dog(s) provide me with benefits (e.g. mental/physical/social/security), so I owe it to them to give them a good life.	Interviews
Dogs have been bred for a certain standard of care so we are obligated to maintain that	Interviews
I owe my dog a happy life	Interviews

I owe my dog fun times	Interviews
I owe my dog unconditional love	Interviews
I owe my dog respect	Interviews
I owe my dog affection	Interviews

Specific

Item	Source
I have a duty to ensure my dog is mentally stimulated	
I have a duty to ensure my dog gets enough exercise	
If my dog were to get sick or injured, I would feel obligated to take them to a vet	
I owe it to my dog to provide preventative health care and veterinary check-ups when they're not sick	
I owe it to my dog to spend time with them everyday	
I owe it to my dog to do research and be prepared in order to care for them correctly	Interviews
I feel obligated to provide my dog with as close to a 'natural life' as possible	Interviews
I owe it to my dog to be plan ahead for their care and wellbeing	Interviews

2. **Ascription of Responsibility:**

General

Item	Source
I feel completely responsible for my dog's health	
I feel completely responsible for my dog's mental state	
I feel completely responsible for my dog's safety	
I am responsible for my dog's behaviour	
My dog is reliant on me to lead a good life	
If my dog was not leading a good life it would be my fault	
I am responsible for my dog's wellbeing	
Dogs depend on their owners for everything	
Someone who does not care for their dog appropriately cannot be held responsible for the consequences of their actions	(Harland et al., 2007)
No matter what the circumstances, dog owners are responsible for their dog's welfare	(Harland et al., 2007)
No matter what the circumstances, dog owners are responsible for their dog's behaviour	(Harland et al., 2007)

Specific

Positively framed

**To what extent do you feel responsible for the following:**	
[Dog's name]'s mental state	1. Not at all responsible<br>2. Partly responsible<br>3. Somewhat responsible<br>4. Very responsible<br>5. Completely responsible
[Dog's name] being happy	
[Dog's name] living a good life	
[Dog's name] being healthy	
[Dog's name] being safe	
[Dog's name] being an appropriate weight	

[Dog's name] being mentally stimulated	
[Dog's name] being free of fear	

Negatively framed

To what extent would you feel responsible for the following:	
If [Dog's name] was depressed	1. Not at all responsible<br>2. Partly responsible<br>3. Somewhat responsible<br>4. Very responsible<br>5. Completely responsible
If [Dog's name] was anxious	
If [Dog's name] was not healthy	
If [Dog's name] was underweight	
If [Dog's name] escaped	
If [Dog's name] engaged in problem behaviours like destroying things or excessive barking	
If [Dog's name] felt insecure or afraid	
If [Dog's name] was bored	
If [Dog's name] was lonely	
If [Dog's name] was aggressive to other people or dogs	

3. **Awareness of Need/Problem awareness:**

<u>General</u>

Item	Source
Providing good care for my dog improves their behaviour	
Problem behaviours in dogs (e.g. destructive behaviours, excessive barking) are often the result of their needs not being met	
Poor dog welfare is a result of owner actions (or inaction)	
Problem behaviours in dogs (e.g. destructive behaviours, excessive barking, aggression) are an indicator of poor welfare	
My dog's welfare could be better	
My dog could be happier	
My dog could be healthier	
My dog could lead a better life than they currently lead	
I am motivated to do particular things for my dog because I know I will have to deal with the consequences if I don't	Interviews
The impact of my behaviour on my dog's welfare is negligible	(Harland et al., 2007)
My dog's welfare is dependent on my actions	(Glanville, 2017)
How I manage my dog affects his/her behaviour	(Glanville, 2017)

<u>Specific</u>

Item	Source
Punishing a dog can lead to further behavioural problems	
Providing toys/puzzles/enrichment items helps keep dogs occupied and not engaging in problem behaviours	
If I don't walk my dog they will be poorly behaved or annoying	Interviews
If my dog does not get enough exercise their welfare will suffer	

If my dog does not get enough mental stimulation their welfare will suffer	
I am motivated to walk/exercise my dog because I know I will have to deal with negative consequences for their behaviour if I don't	Interviews
Dogs that aren't trained are difficult to live with	
If I don't spend time with my dog, they will be sad	

4. **Efficacy**

Item	Source
There are things I could do to make my dog happier	
There are things I could do to make my dog healthier	
There are things I could do to improve my dog's life	

5. **Emotions:**

Item	Source
I would feel guilty if my dog had poor welfare	
It makes me feel proud when I see my dog happy and healthy	Interviews
I would feel ashamed if my dog wasn't getting what they needed	
If I don't walk my dog regularly, I feel guilty	Interviews
If an animal professional (e.g. vet, trainer, inspector) told me that I was not caring for my dog appropriately I would feel ashamed or guilty	
It is important to me that when my dog passes away that I have no regrets regarding how I've treated them	Interviews
I feel bad when I go to work (or would if I worked away from home)	Interviews
Seeing how things that I do (e.g. taking them for a walk) make my dog happy is rewarding for me	Interviews
I do certain things for my dog to alleviate my own guilt	

6. **Standard of Care**

Item	Source
I think that the standard of care that I provide for my dog is higher than most other dog owners	
My dog's wellbeing is always a factor when planning activities or holidays	
Spending time with my dog is important for their wellbeing	
Dogs should be treated like part of the family	
Dogs should be allowed inside	
Dogs should sleep outside	
Dogs should live outside all the time	
Dogs should be given a soft bed to sleep in	
Dogs need to spend time with their owners every day	
Dogs need to be exercised every day	
Dogs should receive regular (e.g. annual) vet check-ups even when they're not sick	

References

BAMBERG, S., HUNECKE, M. & BLÖBAUM, A. 2007. Social context, personal norms and the use of public transportation: Two field studies. *Journal of Environmental Psychology,* 27**,** 190-203.

BENZ-SCHWARZBURG, J., MONSÓ, S. & HUBER, L. 2020. How Dogs Perceive Humans and How Humans Should Treat Their Pet Dogs: Linking Cognition With Ethics. *Frontiers in Psychology,* 11.

GLANVILLE, C. 2017. *The Role of Duty of Care in Companion Dog Care and Management.* BSc (Hons) Honours Thesis, University of Melbourne.

HARLAND, P., STAATS, H. & WILKE, H. M. 2007. Situational and Personality Factors as Direct or Personal Norm Mediated Predictors of Pro-environmental Behavior: Questions Derived From Norm-activation Theory. *Basic & Applied Social Psychology,* 29**,** 323-334.

HENS, K. 2009. Ethical Responsibilities Towards Dogs: An Inquiry into the Dog-Human Relationship. *Journal of agricultural and environmental ethics***,** 3.

RASOOL, F., ZUBERI, N. A., SIDDIQUI, N. U. & MADNI, M. 2019. Using the Theory of Planned Behavior and Norm Activation Model to Understand Individual Energy Conservation Behavior in Karachi, Pakistan. *International Journal of Economic and Environment Geology***,** 93.

-
