# Supplementary material for: Duty of care in companion dog owners: Preliminary scale development and empirical exploration
Source: PLoS One. 2023 May 17;18(5):e0285278. doi: 10.1371/journal.pone.0285278 (PMC10191305; doi:10.1371/journal.pone.0285278)
Supplement: S4 File — (PDF) [file pone.0285278.s004.pdf]

S4 Reworded duty beliefs

These questions relate to our personal sense of duty or moral obligation towards our dogs. A personal duty or moral obligation is a course of action that you feel compelled to take or expect of yourself because of your own personal beliefs of what is right and wrong. This is not based on legal or societal expectations (though they may influence your personal beliefs).

1. When it comes to my dog's care and welfare I feel…

No duty	1	2	3	4	5	6	7	The highest level of duty possible

2. My duty to care for my dog is:

The least important thing in my life	1	2	3	4	5	6	7	The most important thing in my life

The following relate to your personal sense of duty to provide these things, not necessarily the reality of how your dog lives. E.g. your dog may be very happy in practice, but you may not feel it is your personal duty to ensure this.

3. How personally obligated do you feel to make sure your dog is happy?

Not obligated at all	1	2	3	4	5	6	7	Completely obligated

4. How personally obligated do you feel to make sure your dog is healthy?

Not obligated at all	1	2	3	4	5	6	7	Completely obligated

5. How personally obligated do you feel to *manage* your dog's health (including any medical issues) to the best possible standard?

Not obligated at all	1	2	3	4	5	6	7	Completely obligated

6. How personally obligated do you feel to *manage* your dog's behaviour (including any behavioural problems) to the best possible standard?

Not obligated at all	1	2	3	4	5	6	7	Completely obligated

7. How personally obligated do you feel to provide your dog with positive experiences in life?

Not obligated at all	1	2	3	4	5	6	7	Completely obligated

8. How personally obligated do you feel to minimise your dog's negative experiences in life?

Not obligated at all	1	2	3	4	5	6	7	Completely obligated

9. How personally obligated do you feel to ensure your dog leads the best life they possibly can?

Not obligated at all	1	2	3	4	5	6	7	Completely obligated
