# Supplementary material for: Duty of care in companion dog owners: Preliminary scale development and empirical exploration
Source: PLoS One. 2023 May 17;18(5):e0285278. doi: 10.1371/journal.pone.0285278 (PMC10191305; doi:10.1371/journal.pone.0285278)
Supplement: S5 File — (PDF) [file pone.0285278.s005.pdf]

Duty of Care in Companion Dog Owners

Please note, this scale consists of 5 sub-scales which represent distinct aspects of the duty of care model. The items in each subscale can be summed to produce a score for that element but these sub-scale scores are not to be combined. i.e., DO NOT sum all items in the scale for an overall 'duty of care' score.

Key: DB= Duty beliefs (personal norms) PA= Problem Awareness AoI= Awareness of Impact E=Efficacy AR= Ascription of Responsibility

Please indicate to what extent you agree or disagree with the following statements.

		Strongly disagree	Disagree	Somewhat disagree	Neither agree nor disagree	Somewhat agree	Agree	Strongly agree
DB1	I feel a strong personal obligation to ensure my dog is happy							
DB2	I feel a strong personal obligation to ensure my dog is healthy							
DB3	If you bring an animal into your home, it is your duty to make sure they are happy							
DB4	In the same way that people have basic rights, like food, medical care, and housing, so to do dogs.							
DB5	My dog trusts me, so I must live up to that trust							
DB6	My obligations to my dog stem from them being a part of the family							
DB7	We don't have any particular obligations or duties to our dogs							
PA1	My dog could lead a better life than they currently lead							
PA2	My dog could be happier							
PA3	My dog's welfare could be better							

PA4	My dog could be healthier	◯	◯	◯	◯	◯	◯	◯
AoI1	If my dog does not get enough mental stimulation their welfare will suffer	◯	◯	◯	◯	◯	◯	◯
AoI2	How I manage my dog affects his/her behaviour	◯	◯	◯	◯	◯	◯	◯
AoI3	Providing toys/puzzles/enrichment items helps keep dogs occupied and not engaging in problem behaviours	◯	◯	◯	◯	◯	◯	◯
AoI4	If my dog does not get enough exercise their welfare will suffer	◯	◯	◯	◯	◯	◯	◯
AoI5	Providing good care for my dog improves their behaviour	◯	◯	◯	◯	◯	◯	◯
AoI6	AN.Gen_2 Problem behaviours in dogs (e.g. destructive behaviours, excessive barking) are often the result of their needs not being met	◯	◯	◯	◯	◯	◯	◯
E1	There are things I could do to improve my dog's life	◯	◯	◯	◯	◯	◯	◯
E2	There are things I could do to make my dog happier	◯	◯	◯	◯	◯	◯	◯
E3	There are things I could do to make my dog healthier	◯	◯	◯	◯	◯	◯	◯
AR1*	During challenging times like financial hardship, changes in personal circumstances, or when time is limiting, it is reasonable to lower our expectations for dog care and welfare.	◯	◯	◯	◯	◯	◯	◯

**Note: AR1 is agree-disagree, whereas AR2-AR10 are below.*

To what extent would you/do you feel responsible for the following:

		Not at all responsible	Partly responsible	Somewhat responsible	Very responsible	Completely responsible
AR2	If [dog’s name] felt insecure or afraid	◯	◯	◯	◯	◯
AR3	If [dog’s name] was anxious	◯	◯	◯	◯	◯
AR4	If [dog’s name] was depressed	◯	◯	◯	◯	◯

AR5	[dog’s name] ‘s mental state					
AR6	[dog’s name] being free of fear					
AR7	If [dog’s name] was lonely					
AR8	If [dog’s name] engaged in problem behaviours like destroying things or excessive barking					
AR9	If [dog’s name] was bored					
AR10	If [dog’s name] was aggressive to other people or dogs					
